# Implementation outcomes in context: Leadership and measurement based care implementation in VA substance use disorder programs

**DOI:** 10.1186/1748-5908-10-S1-A74

**Published:** 2015-08-20

**Authors:** Elizabeth Gifford, Angela Fuller, Rebecca Stephens, Krystin Matthews, Dominick DePhilippis, Eric Hawkins, James McKay

**Affiliations:** 1 Substance Use Disorder Quality Enhancement Research Initiative, VA Palo Alto Health Care System, 795 Willow Road (MPD 152), Menlo Park, CA 94025, USA; 2 University of Texas School of Public Health, Austin, Texas, 1616 Guadalupe, Suite 6.300, Austin, TX 78701, USA; 3 Department of Psychiatry, University of Pennsylvania Perelman School of Medicine, 415 Curie Blvd, Philadelphia, PA 19104, USA; 4 Philadelphia Center of Excellence in Substance Abuse Treatment and Education, Philadelphia VA Medical Center, 3900 Woodland Avenue, Philadelphia, PA 19104, USA; 5 Department of Psychiatry and Behavioral Sciences, University of Washington School of Medicine, 4333 Brooklyn Ave NE, Seattle, WA 98105, USA; 6 Seattle Center of Excellence in Substance Abuse Treatment and Education, VA Puget Sound Health Care System, 1660 S. Columbian Way, Seattle, WA 98108, USA

## Background

Measurement based care (MBC) involves using standardized instruments to assess patient progress at regular intervals to inform treatment decisions. The aim of this Quality Enhancement Research Initiative (QUERI) project was to characterize context, process, and outcome factors in measurement based care implementation within VA substance use disorder (SUD) programs.

## Methods

We used a mixed-method, multiple case study design to explore implementation of MBC using the nationally supported Brief Addiction Monitor (BAM). Intensive interview and observational data were collected through site visits to six high implementation and two low implementation comparison SUD programs. The pathways these programs took relative to their specific contexts, barriers, and facilitators provided data on implementation process, outcomes, and context. These data informed development of a national survey assessing measurement based care implementation influences and outcomes in SUD program providers. Qualitative and quantitative methods were based on the Promoting Action on Research in Health Sciences (PARIHS) model and Proctor's taxonomy of implementation outcomes.

## Results

Qualitative results identified the important influence of program leaders who engage staff and set structured expectations about measurement based care implementation. Primary barriers were limited time, staff, IT resources, and staff reservations. Providers indicated fewer concerns about barriers when programs took small logistical steps that allowed them to experience that implementation was not overly burdensome. In survey results (N = 148), adoption, acceptability, appropriateness, feasibility, penetration, and fidelity implementation outcomes were significantly related to leadership priority. Relative advantage, compatibility, trialability, simplicity, and provider confidence in use of the BAM were associated with higher fidelity BAM implementation.

## Conclusions

Measurement based care implementation in the complex VA environment highlights the impact of proximal leadership context on implementation outcomes. Consistent, engaging supervisors may help providers gain experience with implementation, directly or indirectly contributing to implementation outcomes by improving providers' confidence, perception of intervention characteristics, and exposure to implementation benefits.

